# Identification and Functional Analysis of the First Aquaporin from Striped Stem Borer, *Chilo suppressalis*

**DOI:** 10.3389/fphys.2018.00057

**Published:** 2018-02-05

**Authors:** Ming-Xing Lu, Dan-Dan Pan, Jing Xu, Yang Liu, Gui-Rong Wang, Yu-Zhou Du

**Affiliations:** ^1^College of Horticulture and Plant Protection and Institute of Applied Entomology, Yangzhou University, Yangzhou, China; ^2^Jiangsu Key Laboratory of Crop Genetics and Physiology, Co-Innovation Center for Modern Production Technology of Grain Crops, Yangzhou University, Yangzhou, China; ^3^State Key Laboratory for Biology of Plant Diseases and Insect Pests, Institute of Plant Protection, Chinese Academy of Agricultural Sciences, Beijing, China

**Keywords:** *Drosophila* integral protein (Drip), *Chilo suppressalis*, structure, expression, functional assay

## Abstract

Aquaporins are integral membrane proteins some of which form high capacity water-selective channels, promoting water permeation across cell membranes. In this study, we isolated the aquaporin transcript (*Cs*Drip1) of *Chilo suppressalis*, one of the important rice pests. *Cs*Drip1 included two variants, *Cs*Drip1_v1 and *Cs*Drip1_v2. Although *Cs*Drip1_v2 sequence (>409 bp) was longer than *Cs*Drip1_v1, they possessed the same open reading frame (ORF). Protein structure and topology of *Cs*Drip1 was analyzed using a predicted model, and the results demonstrated the conserved properties of insect water-specific aquaporins, including 6 transmembrane domains, 2 NPA motifs, ar/R constriction region (Phe^69^, His^194^, Ser^203^, and Arg^209^) and the C-terminal peptide sequence ending in “SYDF.” Our data revealed that the *Xenopus* oocytes expressing *Cs*Drip1 indicated *Cs*Drip1 could transport water instead of glycerol, trehalose and urea. Further, the transcript of *Cs*Drip1 expressed ubiquitously but differentially in different tissues or organs and developmental stages of *C. suppressalis. Cs*Drip1 mRNA exhibited the highest level of expression within hindgut and the third instar larvae. Regardless of pupae and adults, there were significantly different expression levels of *Cs*Drip1 gene between male and female. Different from at low temperature, the transcript of *Cs*Drip1 in larvae exposed to high temperature was increased significantly. Moreover, the mRNA levels of *Cs*Drip1 in the third instar larvae, the fifth instar larvae, pupae (male and female), and adults (male and female) under different humidities were investigated. However, the mRNA levels of *Cs*Drip1 of only female and male adults were changed remarkably. In conclusions, *Cs*Drip1 plays important roles in maintaining water homeostasis in this important rice pest.

## Introduction

Obviously, water is one of the most fundamental molecules for all living organisms. Scientists have confirmed that in addition to simple diffusion, there were high capacity water-selective channels to account for the high water permeability in certain tissues of the animals (Preston et al., 1992; Shakesby et al., 2009). Aquaporins (AQPs), often known as water channels, are integral membrane proteins that regulate the flow of water driven by osmotic gradients (Campbell et al., 2008; Benga, 2009). AQPs exist as tetramers in the cell membrane with each monomer functioning as a water channel (Agre et al., 1987; van Hoek et al., 1991). These AQPs are present in both prokaryotes and eukaryotes species and play an important role in the water transport system (Heymann and Engel, 1999; Agre, 2006; Campbell et al., 2008). Some AQPs can also transport a number of small solutes, typically glycerol or urea (Rojek et al., 2008). Current research on AQPs primarily focuses on plants and vertebrates (Verkman, 2005; Campbell et al., 2008; Maurel et al., 2008). To date, 13 AQP types have been isolated from mammals, named AQP0-AQP12 (Yasui, 2004; Ishibashi, 2006), However, only a few AQP genes were identified and characterized from insects, among which just four species come from Lepidoptera, *Bombyx mori, Grapholita molesta, Spodoptera litura*, and *Ectropis obliqua* (Dow et al., 1995; LeCahérec et al., 1996a,b; Echevarria et al., 2001; Yanochko and Yool, 2002; Duchesne et al., 2003; Kaufmann et al., 2005; Kikawada et al., 2008; Kambara et al., 2009; Kataoka et al., 2009a,b; Shakesby et al., 2009; Goto et al., 2011; Herraiz et al., 2011; Mathew et al., 2011; Philip et al., 2011; Azuma et al., 2012; Liu et al., 2013; Fabrick et al., 2014; Ibanez et al., 2014; Li et al., 2016; Van Ekert et al., 2016; Liu and Piermarini, 2017). The phylogenetic analyses of insect AQPs had revealed the existence of five major subfamilies, including the Drip, Prip, Bib, Eglps, and AQP12L, and members of the Drip and Prip subfamilies typically were water selective channels (Chawn and Nicolson, 2004; Kambara et al., 2009; Herraiz et al., 2011; Mathew et al., 2011; Drake et al., 2015; Finn and Cerda, 2015; Van Ekert et al., 2016). Meantime, according to the database of genome and transcriptome, the only one AQP in *Chilo suppressalis*, two AQPs in *Bombyx mori*, three AQPs in *Danaus plexippus* and *Manduca sexta*, and five AQPs in *Manduca sexta* was identified (http://www.insect-genome.com/data/detail.php?id=7) (Yin et al., 2014), and basing on the homology of AQPs, the above species all could possess the water-selective AQPs. Therefore, Drip and Prip are very important to maintain the balance of water in insect.

Otherwise, some studies suggested that AQPs played an important role in the physiological functions of insect (Goto et al., 2011; Benoit et al., 2014b; Fabrick et al., 2014; Drake et al., 2015). For example, freeze tolerance of insects was related to the ability to remove water from cells by AQPs (Philip et al., 2011). The removal of water from the cells could suspend metabolic processes or avoid damaging ice crystal formation (Spring et al., 2009). Freeze tolerance also needed to accumulate glycerol in the cells, a role admirably suited to the aquaglyceroporins (Spring et al., 2009). Down regulation of AQPs in *Aedes aegypti* enhanced mosquito desiccation resistance (Drake et al., 2015). The female tsetse flies of *Glossina morsitans morsitans* have been studied to elucidate the role of AQPs in heat tolerance (Benoit et al., 2014a,b).

The striped stem borer, *Chilo suppressalis* (Walker) (Insecta: Lepidoptera: Pyralidae), an important rice pest widely distributed in Asia, has caused significant damage to rice crops in China, especially to hybrid rice varieties in recent years. In the district of Yangzhou (32.23°N, 119.26°E), Jiangsu province, China, *C. suppressalis* has two complete and a partial third generation each year, and the larvae enter facultative diapause in winter (Lu et al., 2013). According to our studies, in March of 2010, field-collected larvae could survive at −21^o^C (Lu et al., 2012). Overwintering larvae of *C. suppressalis* could acquire freeze tolerance (Tsumuki and Hirai, 2007). Some studies demonstrated AQPs might play very important role in the cold tolerance of *C. suppressalis* (Izumi et al., 2006, 2007). However, they did not further study any aquaporin gene. It is well-known that *C. suppressalis* need very high humidity condition in the life cycle (Shang et al., 1979; Lu et al., 2014), and AQPs may help to maintain water homeostasis.

Thus, in this paper we firstly described the characteristics of *C. suppressalis* Drip1 (*Cs*Drip1), and assayed the abundance of *Cs*Drip1 in different tissues or organs and developmental stages. Secondly, in order to understand the *Cs*Drip1 regulation under different humidities, the *Cs*Drip1 mRNA levels of different developmental stages and sexes of *C. suppressalis* were investigated under different humidities. Moreover, in order to understand the relationship between *Cs*Drip1 regulation and temperature, we also studied the expression patterns of *Cs*Drip1 mRNA under various temperatures. Last but not the least, to further demonstrate the functions of *Cs*Drip1, functional oocyte swelling assays were executed by water and three kinds of solutes. These studies will help us understand the role of AQPs in *C. suppressalis*, and also may provide insights in developing strategies for the control of this pest.

## Materials and methods

### Insects

The population of *C. suppressalis* was collected from the suburb of Yangzhou (32.39°N, 119.42°E). The rice stem borers were reared in an environmental chamber at 28 ± 1°C, 16:8 (L: D) and RH = 70 ± 5% (Shang et al., 1979).

### Cloning and RACEs

Total RNA of the fifth instar larva was extracted by the SV Total RNA Isolation System (Promega Z3100) combined with DNase digestion to eliminate DNA contamination. Total cDNA was synthesized by oligo(dT)_18_ primer (TaKaRa). Degenerate primers DP-F and DP- R (Table 1) were used to amplify the partial segments of AQP. The full-length cDNA of the *Cs*Drip1 gene was determined using 5′- and 3′-RACE (SMART RACE, Clontech). The primers used (VA and VB) were shown in Table 1. The full length sequence of *Cs*Drip1 was confirmed by the template of RACE 5′ cDNA.

**Table 1:** Primers used in this study.

**Gene**	**Primer name**	**Primer sequences (5′ → 3′)**	**Tm (°C)**	**Length (bp)**
**RT-PCR**				
*Cs*Drip1	F	CACATCAAYCCMGCBGTCAC		431
	R	GGNCCCRCCCARTAMACCCA		
**RACE PCR**				
*Cs*Drip1	3′	CCACGAAGACAGCACCAACCGCAAGA	68.0	666
variant A	5′	CCTTCATGCGGCTAACGTACTCCT	68.0	812
*Cs*Drip1	3′	CCTCCATCACCCGTGCTCGTTTT	68.0	757
variant B	5′	TGGCAGCAGCAGTGGGCTCGTTG	68.0	1093
**FUNCTIONAL ASSAYS**				
*Cs*Drip1	F	TCAACTAGTGCCACCATGAAAACGGATTACGCTGT		774
	R	TCAGCGGCCGCTTAGAAGTCATAGGAGCCGC		
**QPCR**				
*Cs*Drip1	F	GTGGATGAGATGCAGAAAAGGA	59.4	120
	R	AAGCGATGTCAGCACAAAGGT		
H3	F	CTGCACCAAGCACTGGTGGA	56.0	184
	R	TAGCGGCGGACTGGAAACG		
EF1	F	AAAATGGACTCGACTGAACCCC	56.6	137
	R	TCTCCGTGCCAACCAGAAATA		
18S	F	GTGATGGGACGAGTGCTTTTATT	62.5	258
	R	GCTGCCTTCCTTGGATGTGG		
ACTIN	F	AAAGAAACAGCAAAAGTCGGGG	56.0	243
	R	GTTCAATGGAGGTTCGGTAAGTAAA		
TUB	F	GAGGGCATGGACGAGATGGA	60.4	178
	R	ACGACGGTACGAGTATGACGGG		
UBI	F	TCACCGACAGCAAACCAGACT	60.2	219
	R	GGAAGAAAACACCCCCCTCATATA		

*The qPCR primers used in this study were validated*.

### Sample preparation

The rice stem borers were reared successively to the third generation in the seedlings. Then, the egg masses, the first, second, third, fourth, fifth instar larvae, pupae (male and female), and 1-day adults (male and female) were randomly selected for the experiment. Each experiment was repeated four times. The fifth instar deep diapausing larvae of *C. suppressalis* collected from rice stubble were anesthetized on ice before dissection. Heads (HE), epidermis (EP), fat body (FB), foregut (FG), midgut (MG), hindgut (HG), Malpighian tubules (MT) and haemocytes (HC) were collected and rinsed with 0.9% NaCl. The samples were frozen immediately in liquid nitrogen and stored at −70°C until the experiment.

### Temperature stress treatment

The larvae used in experiments were all 5th instars of similar body size and were assigned randomly to each experimental group. Each group of larvae was confined individually in glass tubes (relative humidity, 90 ± 10%) and exposed to a given temperature (including −11, −9, −3, 0, 27, 36, 39, and 42°C) for 2 h in a constant temperature incubator (DC-3010, Jiangnan equipment). The larvae were recovered at 27 ± 1°C for 2 h, and the survived larvae were frozen in liquid nitrogen and then stored at −70°C.

### Humidity treatment

The third instar larvae, fifth instar larvae, pupae (male and female) and adults (male and female) were treated in humidity chamber (HTC-100, SANTN, Shanghai, China) at 27°C, respectively under four different relative humidity levels (RH) (25, 50, 75, and 95%) for 24 h, and additionally the third instar larvae were treated for 12 h. Each treatment was replicated 30 times, and each replicate consisted of one insect. The humidities chosen were based on a prior pilot experiment. At the same time, survival in each treatment was assessed, and the survived larvae were treated as above.

### Functional oocyte swelling assays

The vector construction followed previously reported protocol (Chang et al., 2015). Firstly, the entire coding region of *Cs*Drip1 was amplified with a high-fidelity polymerase (PrimeSTAR®, HS DNA polymerase. TaKaRa, Tokyo, Japan) using primers with Kozak sequence and restriction enzyme cutting sites (SpeI and NotI). And the PCR products were digested with SpeI and NotI, and subcloned into pT7Ts vector (Invitrogen, Carlsbad, California, USA), and then plasmids were fully linearized with SmaI. The cRNAs of *Cs*Drip1 were synthesized *in vitro* using mMESSAGE mMACHINE T7 kit (Ambion, Austin, TX, USA). Purified cRNAs were resuspended in nuclease-free water at a concentration of 0.2 μg/μl and stored at −80°C.

Unfertilized stage V and VI oocytes of *Xenopus* were defolliculated with 2 mg/ml collagenase I (GIBCO, Carlsbad, CA) in washing buffer (96 mM NaCl, 2 mM KCl, 5 mM MgCl_2_, and 5 mM HEPES [pH 7.6]) for about 1 h at room temperature (26°C). After being cultured overnight at 18°C, oocytes were microinjected with 27.6 nl *Cs*Drip1 cRNA (5.52 ng) and 27.6 nl nuclease-free water as control. After injection, oocytes were incubated for 3 days at 18°C in 1X Ringer's solution (96 mM NaCl, 2 mM KCl, 5 mM MgCl2, 0.8 mM CaCl_2_, and 5 mM HEPES [pH = 7.6]) supplemented with 5% dialysed horse serum, 50 mg/ml tetracycline, 100 mg/ml streptomycin and 550 mg/ml sodium pyruvate.

Osmotic water permeability (*P*_f_) was measured as previously described (Kataoka et al., 2009a,b). Oocytes was transferred to a 3-fold dilution of 1X Ringer's solution with distilled water and images were acquired of the oocyte silhouette every 15 s through a CCD camera DP-72 (Olympus, Tokyo, Japan) attached to a Olympus SZX16 stereomicroscope up to 5 min. The osmotic water permeability (*P*_f_) was calculated as in previous reports (Zhang et al., 1990; Preston et al., 1992; Kataoka et al., 2009a,b) by the following equation: *P*_f_ = V_0_×*d*(V/V_0_)/*dt*/[S × *V*_w_×(Osm_in_-Osm_out_)] where *V*_0_ is the oocyte initial volume (*V*_0_ = 9 × 10^−4^ cm^3^), *S* is the oocyte surface area (*S* = 0.045 cm^2^), *V*_w_ is the molecular volume of the water (*V*_w_ = 18 cm^3^/mol) and Osm_in_ is 202 mmol·kg^−1^ and Osm_out_ is 59 mmol·kg^−1^. Relative oocyte volume (*V*/*V*_0_) was calculated from the relative area (*A*/*A*_0_) in the focal plane, *V*/*V*_0_ = (*A*/*A*_0_)^3/2^.

Oocytes were transferred in an isotonic solution which containing 140 mM of solutes (glycerol, trehalose or urea) for solutes transport assays. To maintain the osmotic equilibrium, the increase in oocyte volume corresponds to the water influx accompanying the solute uptake. The volume changes were recorded for 5 min in the same way as described above. Apparent solute permeability was calculated from the equation: *P*sol = [*d*(*V*/*V*_0_)/*dt*] × (*V*_0_/*S*) (LeCahérec et al., 1996a,b; Duchesne et al., 2003). Water and solute permeabilities were performed at least for nine different *Xenopus* oocytes.

### Quantitative real-time PCR (qPCR) analysis

Total RNA was extracted by the SV Total RNA Isolation System (Promega Z3100), followed by DNase treatment to eliminate DNA contamination. The integrity of the RNA in all samples was verified by comparing the ribosomal RNA bands in ethidium bromide-stained gels. RNA sample purity was estimated using spectrophotometric measurements at 260 and 280 nm (Eppendorf BioPhotometer plus). Total RNA (500 ng) was reverse-transcribed into first-strand cDNA using the SuperScript II reverse transcription (RT)-PCR kit (Bio-Rad). The volume of reaction mixture was 20 μl. Each reaction mixture contained 10 μl of iTaq Universal SYBR Green supermix (2x) (Bio-Rad), 1 μl of each of gene specific primers (Table 1), 2 μl of cDNA templates, and 6 μl ddH_2_O. Reactions were carried out on a CFX-96 real-time PCR system (Bio-Rad). The amplification efficiencies of the target and reference genes were similar in this study. Therefore, the quantity of *Cs*Drip1 mRNA was calculated using the 2^−ΔΔCt^ method (Nolan et al., 2006; Schmittgen and Livak, 2008; Bustin et al., 2009). Relative expression levels of *Cs*Drip1 in different tissue or organs were normalized with histone 3 (*H3*), elongation factor 1 (*EF1*) for different developmental stages and temperature stress (Xu et al., 2017). And previous tests of stability of the reference gene demonstrated *18S rRNA* for the third larvae, *ACTIN* for the fifth larvae, *TUB* for the male pupae, UBI for the female pupae and male adults, and *EF1* for the female adults under different humidity was most suitable respectively, and corresponding reference genes were selected to normalize (data not shown). Following qPCR, the homogeneity of the PCR products was confirmed by melting curve analysis, which was read 5 s per 0.5°C, increment from 65°C to 95°C. Every treatment included four biological replicates, and every repeat was run in triplicate.

### Bioinformatic analysis

The open reading frames (ORFs) were identified with the aid of the ORF Finder software (http://www.ncbi.nlm.nih.gov/gorf/gorf.html). The deduced amino acid sequences were aligned using ClustalX software. Sequence analysis tools of the ExPASy Molecular Biology Server of Swiss Institute of Bioinformatics, including Translate, Compute pI/MW, Blast and TMHMM, were used to analyze the deduced *Cs*Drip1 protein sequence. Phosphorylation and kinases sites were predicted using NetPhos 2.0 and NetPhosK 1.0, respectively (Blom et al., 1999, 2004). Amino acid sequences were used to estimate phylogeny with the neighbor-joining, minimum evolution, maximum likelihood and maximum parsimony methods. Phylogenetic trees were constructed with 1000 bootstrap replicates using MEGA version 7.0 (Kumar et al., 2016).

### Computational molecular modeling

Homology models were generated using Protein Homology/analogy recognition engine V 2.0 (http://www.sbg.bio.ic.ac.uk/~phyre2/html) (Kelley and Sternberg, 2009). Briefly, the *Cs*Drip1 amino acid sequence was aligned by the Phyre2, and the best model of bovine AQP1 X-ray A^*^ structure (PDB ID: 1J4N) was used for modeling analyses (Sui et al., 2001). The Chimera Tool was used to visualize the three-dimensional coordinates for the atoms of the model (Pettersen et al., 2004).

### Statistical analysis

The data were tested for normality using the Shapiro-Wilk's test. Homogeneity of variances among different groups was evaluated by the Levene test. All the data was log-transformed when necessary. Then, the significance of differences between treatments was identified with either a Tukey's test (Homogeneity of variances) or Dunnett's T3 test (Non-homogeneous) for multiple comparisons. In the experiments with two groups, significant differences were determined by Student's *t*-test. The data were analyzed using SPSS16.0 software, and denoted as means ± SE (standard error) (Pallant, 2005).

## Results

### Isolation, cloning, sequencing, and structure of *Cs*Drip1

**Figure 1:**
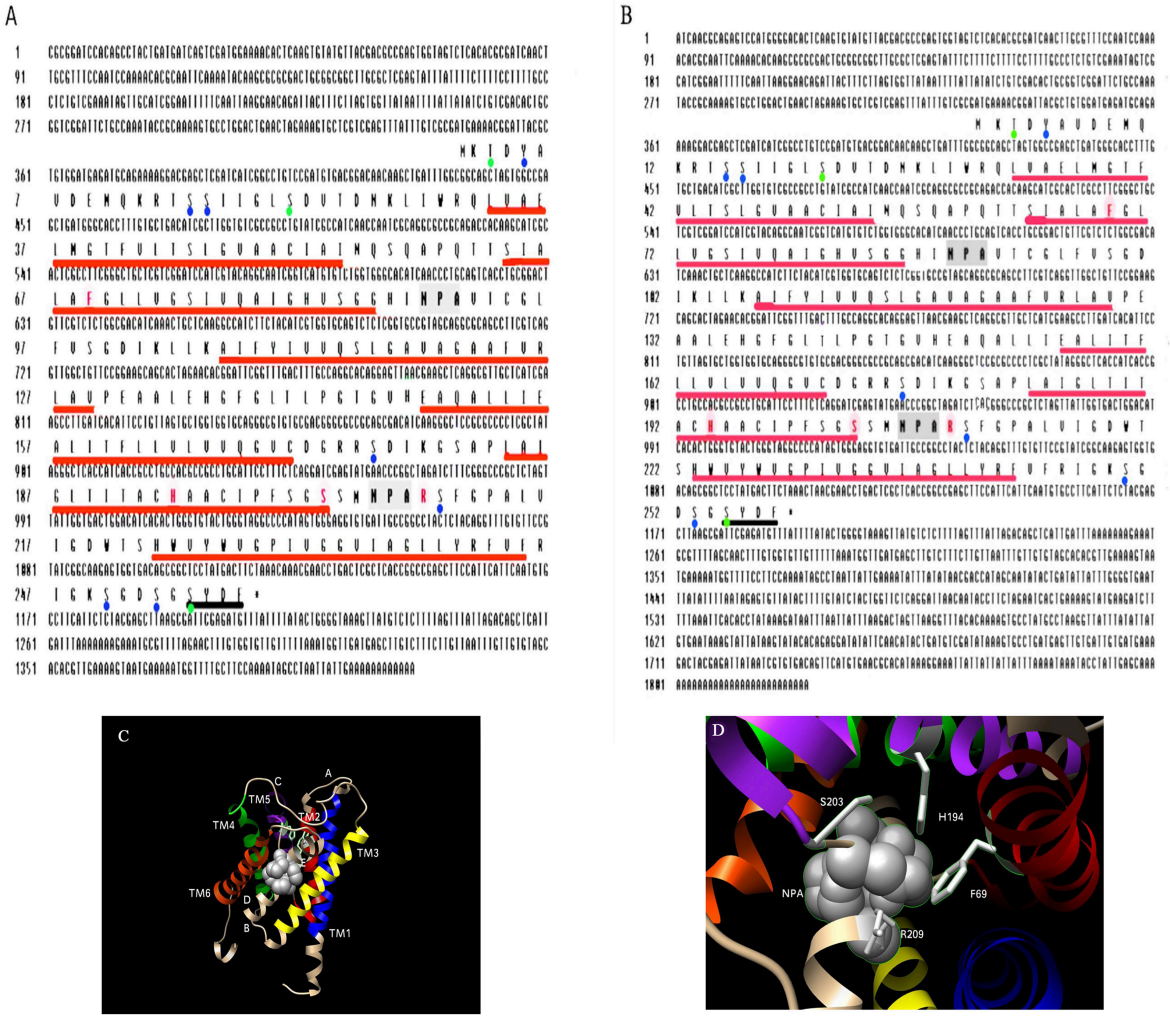
Sequence analysis, predicted topology and structure of *Cs*Drip1. **(A,B)** Nucleotide and deduced amino acid sequence of *Cs*Drip1 variant A (JQ011314) and variant B (JQ011315). Six transmembrane domains (TMs) are also shown by the red underline and C-terminal “SYDF” sequence found in insect AQPs with dark underline. The NPA boxes are shown with gray highlight. Orange indicates amino acids residues constituting the ar/R constriction region. Blue dots and green dots, respectively are potential phosphorylation sites and protein kinase C-specific sites. **(C)** Homology modeling of the *Cs*Drip1 is compared with bovine aquaporin-1 (PDB ID: 1J4N) as template using Phyre2. Intracellular (B,D) and extracellular (A,C,E) loops and transmembrane helices are shown (TM1 in blue, TM2 in red, TM3 in yellow, TM4 in green, TM5 in purple, TM6 in orange). **(D)** The structure depicted is from the extracellular side of the membrane. The classical NPA motifs are shown in gray sphere, and Ar/R selectivity residues regions (Phe-69, His-194, Ser-203, and Arg-209) are shown in gray stick.

Degenerate primers based on conserved regions from several insect aquaporins were used to amplify a 431 bp partial fragment from *C. suppressalis* cDNA. The cloned fragment was sequenced, and a BLAST analysis of its deduced amino acid fragment revealed apparent sequence homology with insect Drip1 (data not shown). The 1,416 and 1,825 bp full-length of variant A and variant B, including the UTRs was obtained, respectively through 5′and 3′ RACE (GenBank accession no. JQ011314 and JQ011315). Two Drip1 types of *C. suppressalis* (*Cs*Drip1_v1 and *Cs*Drip1_v2) were obtained in the present study. Variant A and variant B possessed the same open reading frame (ORF) of 258 amino acids with a predicted molecular weight of 26.9 kDa and an isoelectric point of 6.5. The protein sequence that we referred to as *Cs*Drip1 possessed the hallmarks of the aquaporin family, including the classical “NPA” boxes (residue 89–91; residue 206–208) and 6 putative transmembrane regions (Figures 1A,B). According to the hydropathy analysis, *Cs*Drip1 contained cytoplasmic N- and C-terminal domains and the C-terminus ended with “SYDF”. In the amino acid sequence, seven potential phosphorylation sites (Tyr^5^, Ser^15^, Ser^16^, Ser^176^, Ser^210^, Ser^250^, and Ser^253^) and three potential protein kinase C-specific sites (Thr^3^, Ser^21^, Ser^255^) were also identified (Figures 1A,B). To investigate the potential structure-function relationship of *Cs*Drip1, we generated its homology model with Phyre using bovine aquaporin-1 (1J4N) as a template (Sui et al., 2001). The structure of *Cs*Drip1 model was very similar to that of aquaporin-1 (confidence, 100% statistically and identity, 36% based on sequence alignment). *Cs*Drip1 models showed two tandem structural repeats, each consisting of three transmembrane helices (TM1-3 and TM4-6) and a short α-helix in loops B and E, each containing an NPA motif predicted to line one side of the pore (Figure 1C), which was called the “aquaporin fold” (Murata et al., 2000). Conserved NPAs formed the canonical structure in the center of the pore (Fu et al., 2000), and the structural model provided the defining force that orients water as it passed through the midpoint of the channel (Hoa et al., 2009). Residues that comprised the Ar/R constriction (Phe^69^, His^194^, Ser^203^, and Arg^209^) were found in *Cs*Drip1 and predicted to establish water selectivity (Figures 1C,D).

### Phylogenetic analysis

**Figure 2:**
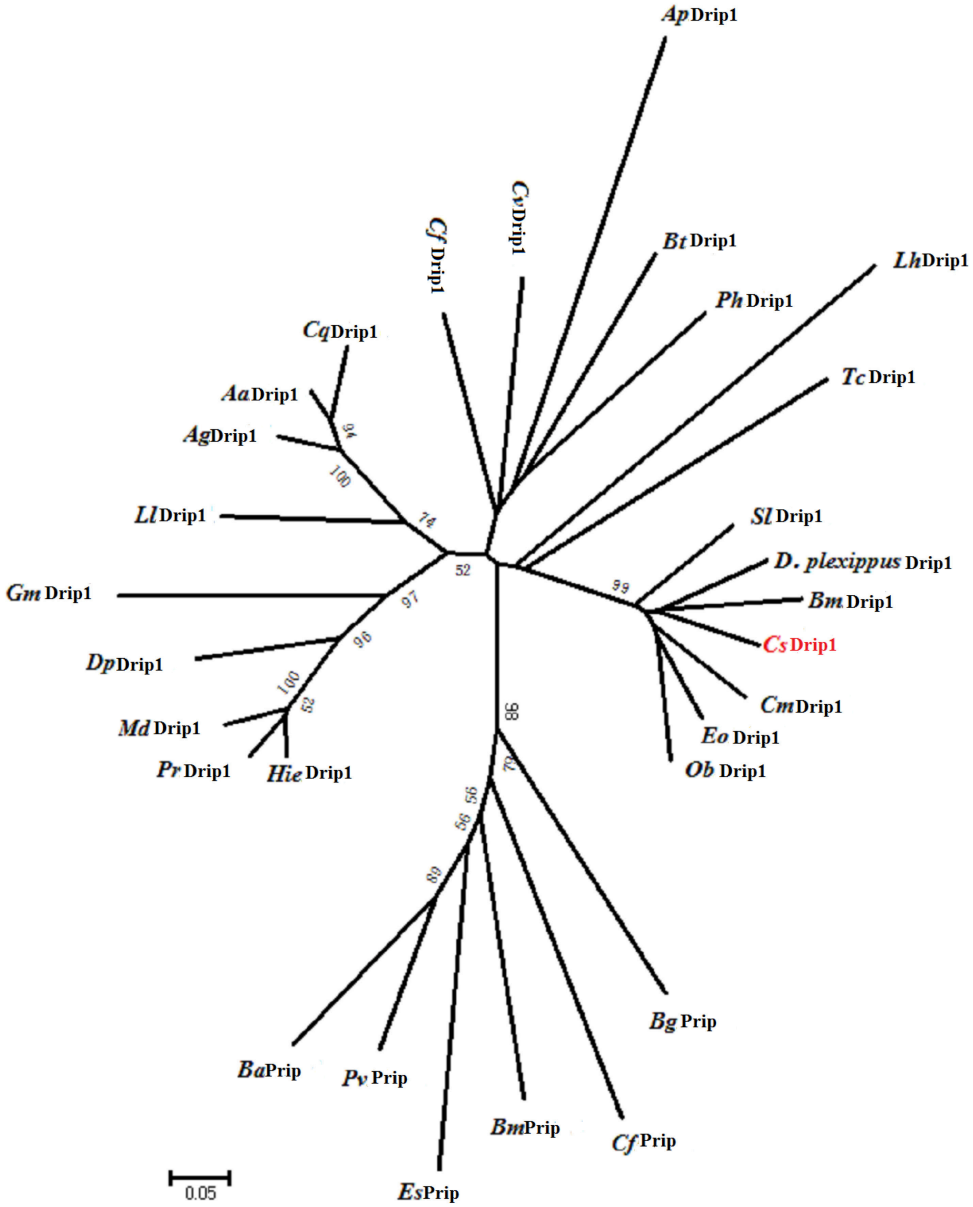
Neighbour-joining phylogenetic tree showing the *Chilo suppressalis* Drip1 (JQ011314 and JQ011315) with respect to other insect AQPs. *Bm*Drip1 (*Bombyx mori*, AB178640), *Cm*Drip1 (*Cydia molesta*, AB469882), *Aa*Drip1 (*Aedes aegypti*, AF218314), *Ph*Drip1 (*Pediculus humanus*, DS235154), *Tc*Drip1 (*Tribolium castaneum*, CM000285), *Ll*Drip1 (*Lutzomyia longipalpis*, EU124628), *Ag*Drip1 (*Anopheles gambiae*, AAAB01008984), *Cf* Drip1 (*Coptotermes formosanus*, AB433197), *Hie*Drip1 (*Haematobia irritans exigua*, U51638), *Cv*Drip1 (*Cicadella viridis*, X97159), *Dp*Drip1 (*Drosophila pseudoobscura*, CM000071), *Bt*Drip1 (*Bemisia tabaci*, EU127479), *Gm*Drip1 (*Glossina morsitans*, EZ422826), *Cq*Drip1 (*Culex quinquefasciatus*, DS232465), *Ap*Drip1 (*Acyrthosiphon pisum*, EU925136), *D. plexippus*Drip1 (*Danaus plexippus*, EHJ75085), *Eo*Drip1 (*Ectropis oblique*, AMO02270.1), *Sl*Drip1 (*Spodoptera litura*, AGT50942.1), *Ob*Drip1 (*Operophtera brumata*, KOB73271.1), *Pr*Drip1 (*Phormia regina*, BAM26200.1), *Md*Drip1 (*Musca domestica*, AIB09141.1), *Lh*Drip1 (*Lygus hesperus*, AHI85743.1), *Es*Prip (*Eurosta solidaginis*, FJ489680), *Bg*Prip (*Blattella germanica*, FR744897), *Cf* Prip (*Camponotus floridanus*, GL435707), *Ba*Prip (*Belgica Antarctica*, AB602341), *Pv*Prip (*Polypedilum vanderplanki*, AB281619), and *Bm*Prip (*Bombyx mori*, AB458833). Numbers on the branches are the bootstrap values obtained from 1,000 replicates (only bootstrap values >50 are shown).

We used CLUSTALX and MEGA 7.0 phylogenetic analysis to compare *Cs*Drip1 with other insect aquaporins. Because neighbor-joining, minimum evolution, maximum likelihood, and maximum parsimony methods constructed the similar phylogenetic tree, Figure 2 only illustrated the phylogenetic tree constructed by the neighbor joining method. The result exhibited the phylogenetic tree include two clusters: Drip1 and Prip. And *Cs*Drip1 (*C. suppressalis* Drip1) was most closely related to *Bm*Drip1 (*B. mori* Drip1) and *Dp*Drip1 (*Danaus plexippus* Drip1), to which they were 79% identical at the amino acid level. Our phylogenetic tree showed *Bm*Drip1, *Dp*Drip1, and *Cs*Drip1 belonged to the same group, which was water-specific Drip subfamily. All the Lepidoptera except *Bm*Prip (*B. mori* Prip) fell into the well-supported cluster, which was consistent with a previous publication about *Bm*Prip classification in the Prip subfamily (Azuma et al., 2012).

### Functional assay

**Figure 3:**
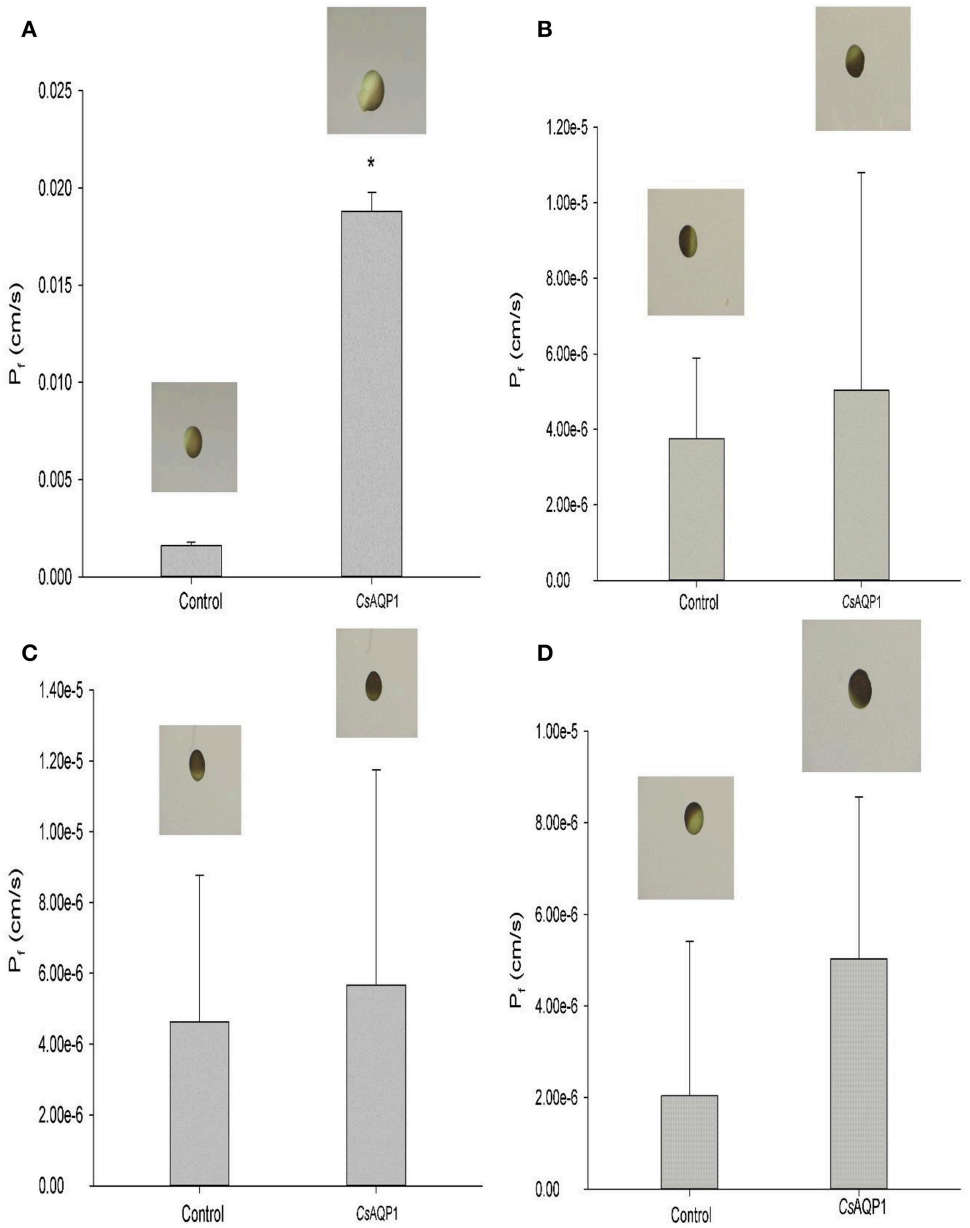
Functional assay of *Chilo suppressalis* aquaporin (*Cs*Drip1). **(A)** Water permeability of *Cs*Drip1. **(B)** Glycerol permeability of *Cs*Drip1. **(C)** Trehalose permeability of *Cs*Drip1. **(D)** Urea permeability of *Cs*Drip1. The graphs of oocytes assayed in the experiments were indicated above each bar. Error bars represented SEM (*n* ≥ 9). Student's *t*-test was performed to find means that were significantly (*p* < 0.05) different from the water-injected control oocytes (indicated with asterisk).

In order to confirm further that *Cs*Drip1 was a water-selective aquaporin, the permeability of *Cs*Drip1 to water, glycerol, trehalose and urea transport was performed using the *Xenopus* oocyte expression system, respectively. Compared to control oocytes, oocytes expressing *Cs*Drip1 showed an 11-fold increase in the osmotic water permeability coefficient (*P*_f_) (*N* = 10) (Figure 3A), and as a result of continuous uptake, even some tested oocytes broke. For glycerol, trehalose and urea uptake in *X. laevis* oocytes expressing *Cs*Drip1, no significant (*p* > 0.05) uptakes were observed in oocytes expressing *Cs*Drip1, and these oocytes showed a slight shrinkage. For example, the *P*_f_ of *Cs*Drip1 oocytes in glycerol, trehalose and urea was 5.031e^−6^ ± 5.766e^−7^ cm/s (*N* = 9), 5.670e^−6^ ± 6.0758e^−7^ cm/s (*N* = 10), and 5.029e^−6^ ± 3.542e^−7^ cm/s (*N* = 11), respectively (Figures 3B–D). These results clearly indicated that *Cs*Drip1 was a specific water-selective aquaporin.

### Expression of *Cs*DRIP1 in tissues or organs of *C. suppressalis* larvae

**Figure 4:**
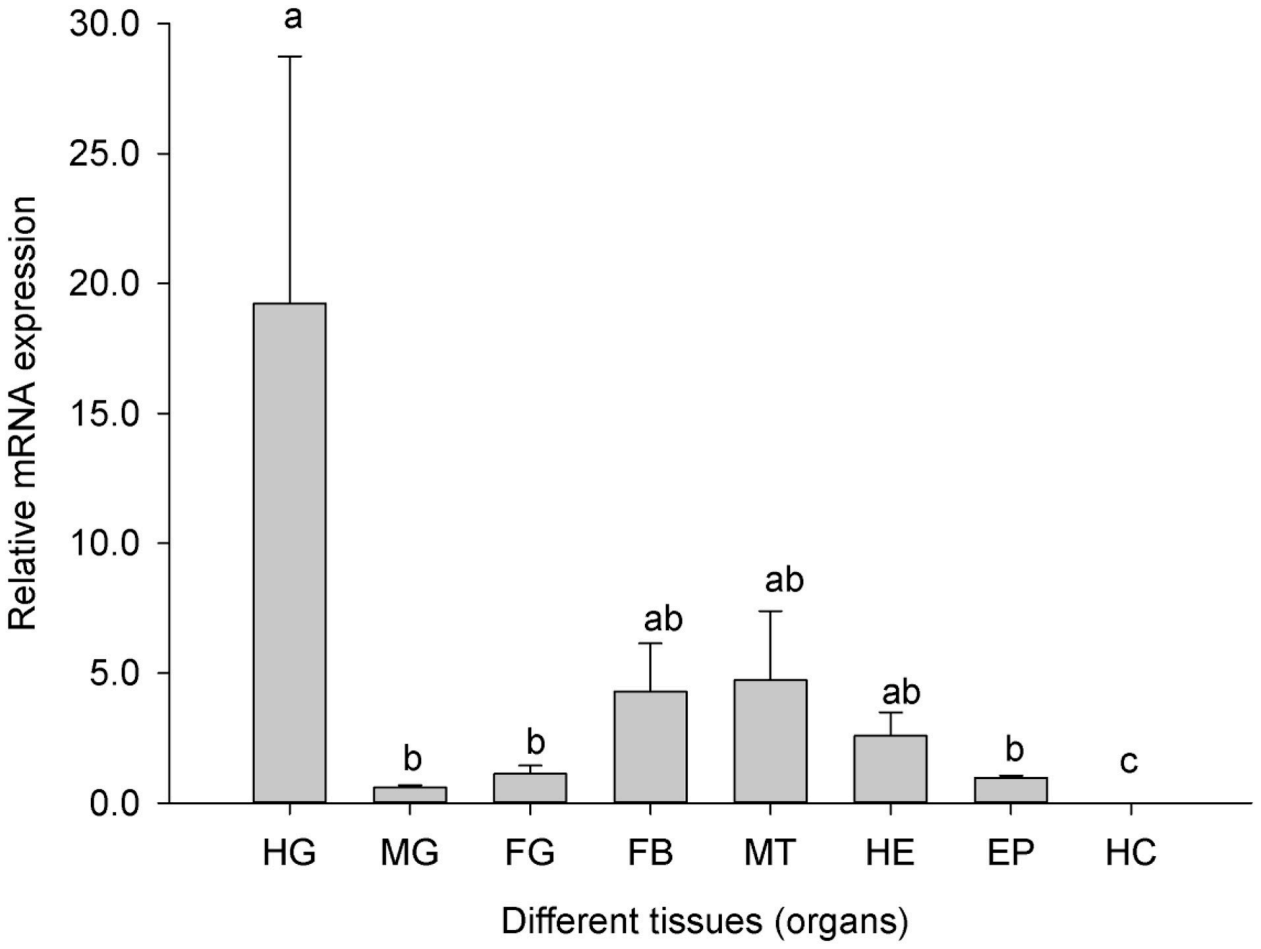
Relative mRNA expression levels of the *Cs*Drip1 gene in different tissues (organs) of the *Chilo suppressalis*. HG, hindgut; MG, midgut; FG, foregut; HC, haemocytes; MT, Malpighian tubules; EP, epidermis; FB, fat body and HE, Heads. Values are denoted as the mean ± SE. Significant differences are indicated as *P* < 0.05.

Real-time PCR verified that *Cs*Drip1 mRNA was expressed in head, epidermis, fat body, foregut, midgut, hindgut, and Malpighian tubules [*F*_(6, 14)_ = 4.441, *P* = 0.010], but there were not expression level in haemocytes. The highest level of *Cs*Drip1 mRNA was observed within hindgut, which was 19.23-fold higher than that in the foregut. And *Cs*Drip1 mRNA in Malpighian tubules also exhibited the second high abundance. Interestingly, the head was one of the organs that expressed a high level of *Cs*Drip1 mRNA (Figure 4).

### Expression of *Cs*DRIP1 in developmental stages of *C. suppressalis*

**Figure 5:**
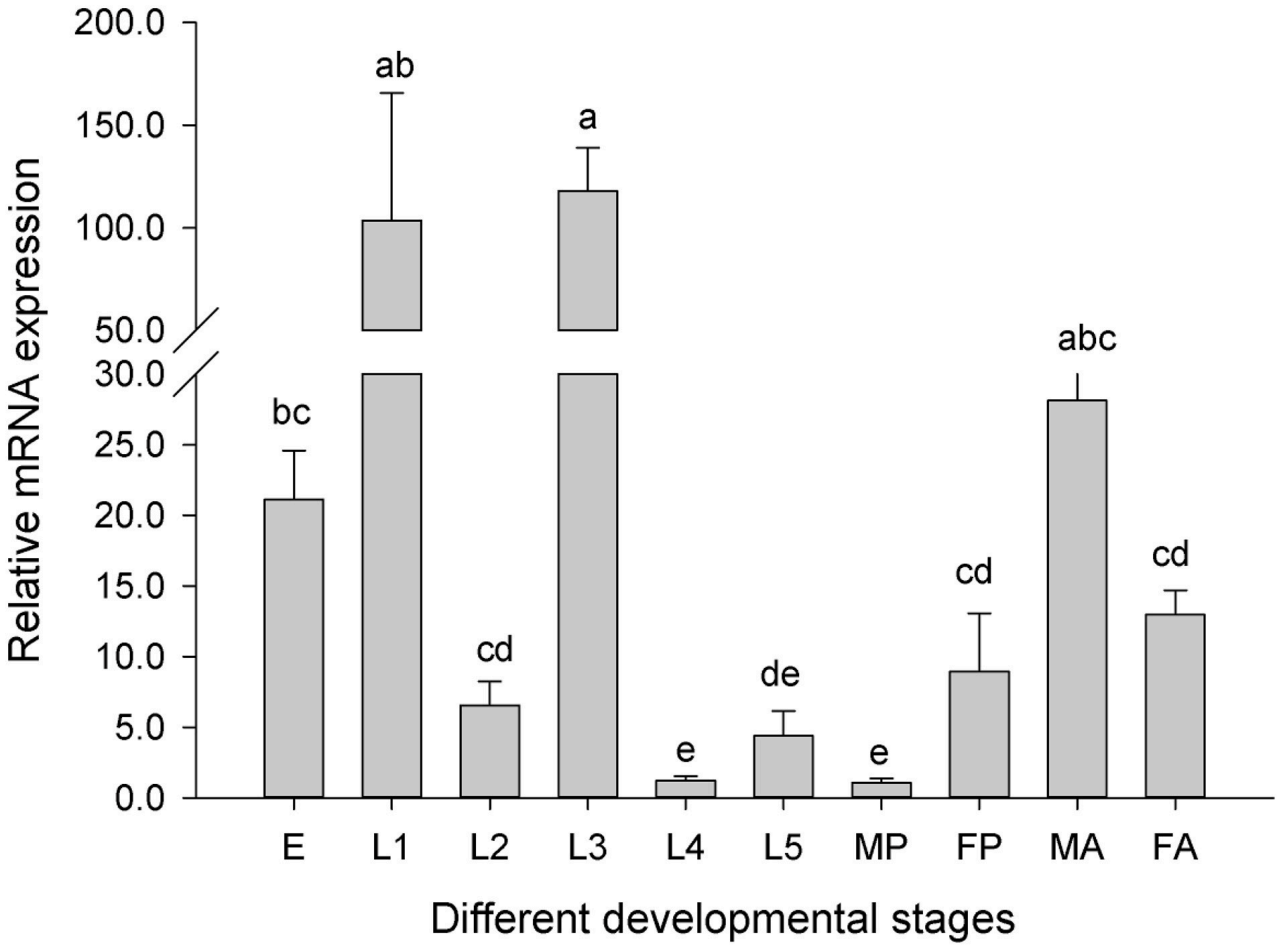
Relative mRNA expression levels of the *Cs*Drip1 gene in different developmental stages of the *Chilo suppressalis*. E, eggs; L1, the first instar larvae; L2, the second instar larvae; L3, the third instar larvae; L4, the fourth instar larvae; L5, the fifth instar larvae; MP, male pupae; FP, female pupae; MA, male adults; and FA, female adults. Values are denoted as the mean ± SE. Significant differences are indicated as *P* < 0.05.

We investigated the expression patterns of *Cs*Drip1 transcripts over the life cycle of *C. suppressalis*, including eggs, larvae (1st, 2nd, 3rd, 4th, and 5th instars larvae), pupae (male and female) and adults (male and female). The results demonstrated that the highest mRNA level of *Cs*Drip1 was observed in the third instar larvae, which was 117.89-fold higher than that of male pupae, which followed by the first instar larvae, male adults, and egg mass. *Cs*Drip1 transcript of the male pupae was the least. In addition, *Cs*Drip1 mRNA exhibited significantly different level in male pupae and male adults than those of female pupae and female adults, respectively [*F*_(9, 19)_ = 23.954, *P* < 0.001] (Figure 5).

### Expression of *Cs*Drip1 under various temperatures

**Figure 6:**
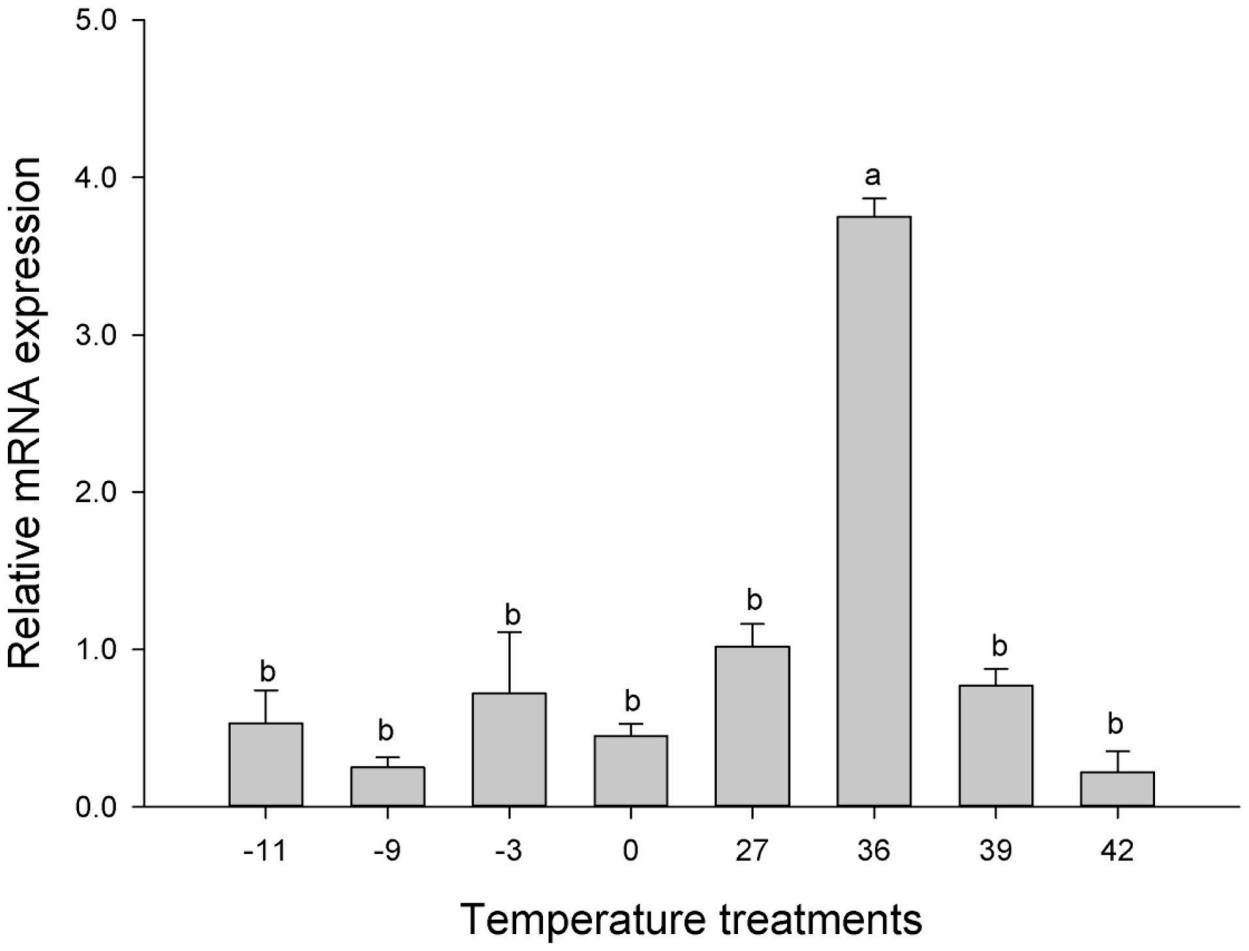
Relative expression levels of *Cs*Drip1 gene in the *Chilo suppressalis* under temperature stress. The larvae were exposed for −11°C, −9°C, −3°C, 0°C, 27°C, 36°C, 39°C, or 42°C for 2 h. Values are denoted as the mean ± SE. Significant differences are indicated as *P* < 0.05.

Although the survival rate of larvae of *C. suppressalis* only reached to 55.56% at −11°C and100% at 42°C, respectively (Lu et al., 2014), *Cs*Drip1 mRNA in larvae exposed to low temperatures for 2 h was not different significantly [*F*_(7, 16)_ = 39.957, *P* = 0.560] (Figure 6). However, *Cs*Drip1 displayed a different expressional pattern under heat stress. For example, *Cs*Drip1 mRNA reached maximum at 36°C, which was 3.75-fold of that at control temperature (27°C) [*F*_(7, 16)_ = 39.957, *P* < 0.001]. Subsequently, the abundance of *Cs*Drip1 mRNA in larvae decreased by the elevated temperature (Figure 6).

### Expression of *Cs*Drip1 in developmental stages of *C. suppressalis* under different humidities

**Figure 7:**
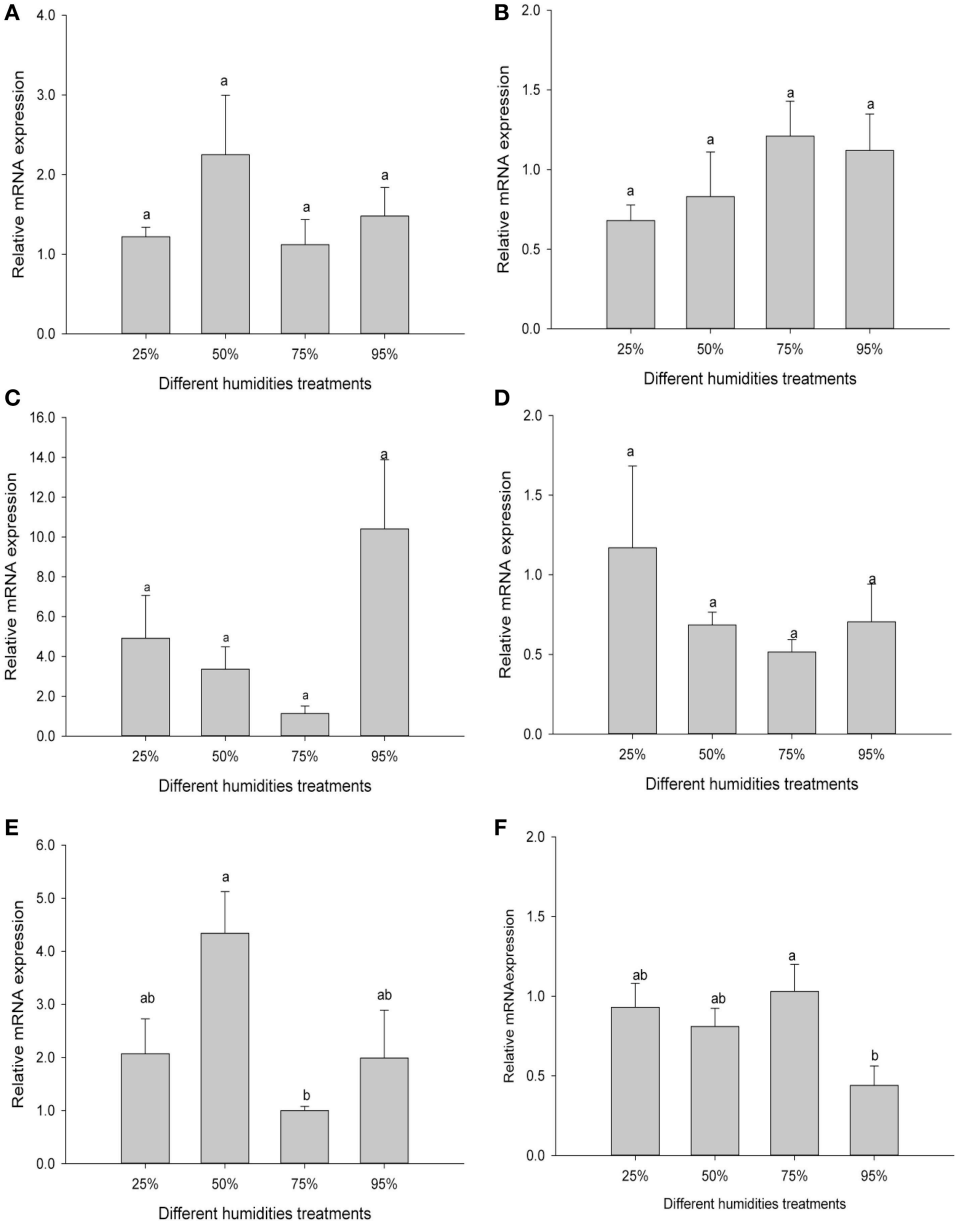
Relative expression levels of *Cs*Drip1 gene in the *Chilo suppressalis* under humidity treatments. **(A)** The third instar larvae treated by 25, 50, 75, and 95% for 12 h, respectively. **(B)** The fifth instar larvae treated by 25, 50, 75, and 95% for 24 h, respectively. **(C)** The female pupae treated by 25, 50, 75, and 95% for 24 h, respectively. **(D)** The male pupae treated by 25, 50, 75, and 95% for 24 h, respectively. **(E)** The female adults treated by 25, 50, 75, and 95% for 24 h, respectively. **(F)** The male adults treated by 25, 50, 75, and 95% for 24 h, respectively. Values are denoted as the mean ± SE. Significant differences are indicated as *P* < 0.05.

According to prior experiments, *C. suppressalis* could tolerate these humidity treatments. The expression levels of *Cs*Drip1 mRNA of the third instar larvae, fifth instar larvae, pupae (male and female) and adults (male and female) of *C. suppressalis* under different humidities have been determined. The results demonstrated that after exposure of the third instar larvae, fifth instar larvae, pupae (male and female) to 25–95% RH, *Cs*Drip1 mRNA levels were not significantly different from each other [*F*_(3, 12)_ = 1.309, *P* = 0.317; *F*_(3, 8)_ = 1.285, *P* = 0.324; *F*_(3, 8)_ = 3.453, *P* = 0.072; *F*_(3, 8)_ = 0.964, *P* = 0.455] (Figures 7A–D). However, the *Cs*Drip1 mRNA of female and male adults exposed to different humidities was changed significantly, and the *Cs*Drip1 mRNA of female adults was up-regulated remarkably, which was contrary to male adults [*F*_(3, 8)_ = 4.272, *P* = 0.045; *F*_(3, 10)_ = 3.816, *P* = 0.047] (Figures 7E,F).

## Discussion

Water-selective aquaporins are integral membrane proteins belonging to a large family of water channel proteins that assist in rapid movement of water across cellular membranes. This report was the first extensive analysis of Drip1 and its functions in *C. suppressalis*, an important rice pest of Lepidoptera. We isolated and characterized the first AQP from the *C. suppressalis* (*Cs*Drip1). Deduced amino acid sequence of *Cs*Drip1 resembled the features of other insect Drip1 (Ibanez et al., 2014). For example, two inverted hemi-helices on loops B and E that project opposing NPA motifs, which regulate the conductance of water (Wree et al., 2011). However, some insect AQPs lacked the first NPA motif, lost the ability to transport water but possessed the capacity of the glycerol transporter (Liu et al., 2016). Additionally, it had seven potential phosphorylation sites (Tyr^5^, Ser^15^, Ser^16^, Ser^176^, Ser^210^, Ser^250^, and Ser^253^), and three potential protein kinase C (PKC) specific sites (Thr^3^, Ser^21^, and Ser^255^) which somewhat contrasted to those of *B. mori* Drip1 (*Bm*Drip1) that had only one PKC specific sites (Thr^6^) and one tyrosine kinase phosphorylation site (Tyr^250^) (Kataoka et al., 2009a). Although *Cs*Drip1 variant A and variant B possessed the same ORF, the full-length sequence of variant A was 409 bp shorter than variant B (Figures 1A,B). Similar phenomenon was found in Prip of *Anopheles gambiae*, which included two variants, but two variants encoded different amino acids (Tsujimoto et al., 2013). It was also found that in *Belgica antarctica* there were three types of Prip (variants A-C) derived from the same gene, which was suggested to be due to alternative splicing (Goto et al., 2011). Hydrophobicity and structural prediction indicated that *Cs*Drip1 possessed the conserved feature of water-specific AQPs. The pore of vertebrate AQPs is restricted by four residues (Phe^58^, His^182^, Cys^191^, and Arg^197^) that comprise the Ar/R constriction site (Sui et al., 2001; Horsefield et al., 2008; Hoa et al., 2009). Although the Ar/R constriction residues are generally conserved, Cys^191^ in vertebrate AQP is replaced by serine (Ser^203^ in TM5) in *Cs*Drip1 (Figures 1C,D). Further comparative analysis of the vertebrate and insect AQPs revealed that either alanine or serine in all known insects Drip1 was substituted for Cys^191^ in vertebrate AQP1, and all Drip1 from Lepidoptera had a serine residue.

Aquaporins are most highly expressed in tissues where water movement is frequent and/or physiologically important. *Cs*Drip1 transcripts showed specific expression patterns in various tissues (Figure 4). Water homeostasis in insects is achieved by a two-part system composed of Malpighian tubules and hindgut, and Malpighian tubules are the primary excretory and osmoregulatory organ in insects, analogous to the vertebrate renal tubules (Chawn and Nicolson, 2004). *Cs*Drip1 mRNA was expressed abundantly in hindgut and Malpighian tubules, which was similar to the results observed in several other insects (Kambara et al., 2009; Kataoka et al., 2009a,b; Drake et al., 2010, 2015; Goto et al., 2011; Liu et al., 2011; Nagae et al., 2013; Fabrick et al., 2014). Fat body was one of the most cold-resistant tissues in the *C. suppressalis* because glycerol was accumulated in the fat body of cold acclimated and diapause larvae (Izumi et al., 2006, 2007). And our results revealed that *Cs*Drip1 mRNA in fat body was high (Figure 4). However, low abundance or absence of Drip1 in the fat body had also been observed in several insect species (Kambara et al., 2009; Liu et al., 2011, 2013; Philip et al., 2011). Fat body was the site of glycerol biosynthesis (Kukal et al., 1988), which likely requires other kind of aquaporins, aquaglyceroporins for glycerol transportation as suggested in *B. mori* and *G. molesta* (Kataoka et al., 2009a,b). Therefore, *Cs*Drip1 in the diapausing larvae of *C. suppressalis* couldn't contribute its cold hardiness. In summary, an unexpected diversity of AQPs was found in insect, and different AQPs contributed various functions. For example, there were six AQP genes in *Ae. Aegypti*. However, AQP 1 and AQP 2 were the strict water channels, which raised the of water permeability of midgut and Malpighian tubules. And AQP 5 demonstrated significant solute permeability for trehalose, which was important for insect temperature and dehydration tolerance (Drake et al., 2010, 2015; Van Ekert et al., 2016).

The levels of *Cs*Drip1 transcript are high at early stages of development, and the expression of *Cs*Drip1 was quite dynamic throughout development (Figure 5). *Cs*Drip1 mRNA was highest in the third larvae of *C. suppressalis*, which just entered into the guzzled stage. The eggs of insect generally require high moist environment condition for hatching. High abundance of *Cs*Drip1 mRNA in eggs of *C. suppressalis* was propitious to maintain the water balance of the embryonic development. However, *Bt*Drip1 transcripts were most highly expressed in 2nd instar nymphs and least present in eggs (Mathew et al., 2011). AgDrip1 mRNA was higher in female adults than that in male adults (Liu et al., 2011), whereas *Cs*Drip1 mRNA in male adults was significantly higher than in female adults. In addition, *Cs*Drip1 mRNA was also greater in adults than that in pupae (Figure 5). We speculated that *Cs*Drip1 expression might be related to reproduction.

When exposed to extreme temperatures, insects may respond in different ways: they can adopt behaviors to avoid or escape extreme temperatures, or they may regulate various proteins in response to adverse temperatures (Tursman et al., 1994; Rinehart and Denlinger, 2000; Tyshenko et al., 2005; Huang et al., 2007; Cui et al., 2010). In *E. solidaginis* by increasing the number of AQPs, cells likely improved their ability to rapidly redistribute water, better protecting themselves against the build-up of osmotic pressures across the membrane during freezing in winter (Philip et al., 2008, 2011). In *Megaphorura arctica*, up- or down-regulation of AQPs were contributed to exploit cryoprotective dehydration to enhance its cold tolerance (Clark et al., 2009). The AQPs of *B. Antarctica* also play very important roles in its freeze tolerance (Yi et al., 2011). And the studies suggested that the aquaporin might contribute to the cold tolerance of *C. suppressalis* (Izumi et al., 2006, 2007). The *Cs*Drip1 might play the important role in the cold hardiness and diapause initiation of *C. suppressalis* (Lu et al., 2013). However, the *Cs*Drip1 transcript in our studies was not up-regulated significantly under low temperature stress, but the *Cs*Drip1 transcript increased significantly under high temperature stress (Figure 6). At the same time, our results confirm that *Cs*Drip1 is a strict water channel. Therefore, *C. suppressalis* could utilize the up-regulation of *Cs*Drip1 to exchange water in order to escape heat stress, but survival at low temperature they might largely depend on the ability of cells to accumulate the cryoprotectants among cellular compartments. Maybe, other AQPs existing in *C. suppressalis* could be coordinated to resist to the low stress.

Although the *Xenopus* oocyte expression system clearly demonstrated that *Cs*Drip1 allowed water, but not glycerol, trehalose or urea, to pass through the cell, *Cs*Drip1 mRNA of the third instar larvae, fifth instar larvae, and pupae (male and female) of *C. suppressalis* after exposure to 25–95% RH was not significantly regulated. However, *Cs*Drip1 mRNA in female adults was greatly induced while expression of *Cs*Drip1 mRNA in male adults was suppressed (Figure 7). Our data are inconsistent with that from a previous study in *C. pipiens*, which found that Prip mRNA level was significantly down-regulated in response to a low relative humidity (Liu and Piermarini, 2017). In the ovary of *B. antarctica* under water stress, no significant differences were observed in the levels of *Ba*Prip mRNA (Goto et al., 2011). In response to dehydration, expression of *Pv*Drip1 of *Polypedilum vanderplanki* larvae was greatly induced (Kikawada et al., 2008). Therefore, when confronted with water stress, different kinds of insects could possess different strategies by regulating different AQPs.

It is widely known that most insects possess multiple AQP genes. At least seven putative AQPs in *A. gambia*, six in *Ae. Aegypti*, eight in *Drosophila*, five in *Lygus hesperus*, eight in *Bemisia tabaci*, and seven in *Triboleum castaneum* had been identified (Adams et al., 2000; Holt et al., 2002; Drake et al., 2010; Fabrick et al., 2014; Van Ekert et al., 2016). And three AQPs in *B. mori* and two in *G. molesta* from Lepidoptera also had been isolated (Kataoka et al., 2009a,b; Azuma et al., 2012). The fat body of *C. suppressalis* could contain aquaglyceroporins related to the diapause and cold hardness (Izumi et al., 2006, 2007). Therefore, it's significant to identify and further analyse the other AQPs in *C. suppressalis*. We expect that the research of *Cs*AQPs will be key in revealing the plot underlying mechanism of development, temperature tolerance, diapause and distribution of *C. suppressalis* in the future.

## Author contributions

Y-ZD and G-RW conceived and designed the experiments. M-XL, YL, D-DP, and JX preformed the experiments. M-XL and Y-ZD analyzed the data and wrote the manuscript.

### Conflict of interest statement

The authors declare that the research was conducted in the absence of any commercial or financial relationships that could be construed as a potential conflict of interest.
